# Treatment of patients with hereditary angioedema with the c.988A>G (p.Lys330Glu) variant in the plasminogen gene

**DOI:** 10.1186/s13023-020-1334-8

**Published:** 2020-02-17

**Authors:** Konrad Bork, Karin Wulff, Guenther Witzke, Thomas Machnig, Jochen Hardt

**Affiliations:** 1 Department of Dermatology, Johannes Gutenberg University, Langenbeckstr. 1, 55131 Mainz, Germany; 2 University Medicine, Ernst Moritz Arndt University, Greifswald, Germany; 3 CSL Behring GmbH, Marburg, Germany; 4 Department of Medical Psychology and Medical Sociology, Johannes Gutenberg University, Mainz, Germany

**Keywords:** Hereditary angioedema, Plasminogen, Genetics, Hereditary angioedema with normal C1 inhibitor, Icatibant, Plasma-derived C1-INH treatment

## Abstract

**Background:**

Hereditary angioedema (HAE) in patients with normal C1 inhibitor (C1-INH) and the c.988A > G (p.Lys330Glu; p.K330E) variant in the plasminogen gene (HAE-PLG) is associated with skin swellings, abdominal pain attacks, and the risk of asphyxiation due to upper airway obstruction. Aim of this observational, retrospective study is to report about the efficacy of various treatments for acute attacks and long-term prophylaxis.

**Results:**

The study included 111 patients with HAE-PLG. Thirteen patients were treated with icatibant for 201 acute swelling attacks. The mean duration of the treated attacks (mean 4.3 h; standard deviation [SD] 2.6 h) was significantly shorter than that of the previous 149 untreated attacks (mean 44.7 h; SD 28.6 h, *p* < 0.0001). Twelve patients were treated with plasma-derived C1-INH for 74 acute swelling attacks. The duration of the treated attacks (mean 31.5 h; SD 18.6 h) was significantly shorter than that of the previous 129 untreated in the same patients (mean 48.2 h; SD 32.5 h, *p* < 0.0001). Corticosteroids alone showed good response in 61/268 attacks (8 patients), low response in 82/268 attacks (7 patients), and no response in 125/268 attacks (26 patients). Corticosteroids combined with antihistamines showed good response in 13/309 attacks (4 patients), low response in 150/309 attacks (7 patients), and no response in 146/309 attacks (17 patients). Antihistamines alone were ineffective in all 37 attacks of 5 patients. In 2 patients with imminent asphyxiation due to tongue swelling and partial obstruction of the upper airways fresh frozen plasma was used without clinical response. The mean reduction in attack frequency was 46.3% under progestins (6 patients), 93.9% under tranexamic acid (3 patients) and 83.3% under danazol (3 patients).

**Conclusions:**

For patients with HAE-PLG various treatment options are available, which completely or at least partially reduce attack duration or attack frequency.

## Background

Hereditary angioedema (HAE) is characterized by recurrent localized and self-limited edema episodes in various organs. Clinical symptoms include skin swellings, abdominal pain attacks due to wall edema of the gastrointestinal tract, tongue swellings and laryngeal and pharyngeal edema, potentially causing dyspnoea and death by asphyxiation. Variants in different genes are associated with clinical symptoms of HAE. Thus, various genotypes are leading to the phenotypes of HAE. In 1963 a biochemical C1 inhibitor (C1-INH) deficiency was identified in HAE by Virginia Donaldson as the root cause of HAE (HAE due to C1-INH deficiency, HAE-C1-INH) [1]. The genetic defect in this type of HAE (HAE-C1-INH) in the *SERPING1* gene has been identified in 1987 [2].

In 2000, a new type of HAE was described which was not associated with a deficiency of C1-INH [3, 4]. It was termed “HAE with normal C1-INH” (HAEnCI) or “HAE type III”. Today it is well established that HAEnCI is not linked to the same genetic variant in all families and that HAEnCI is not a single HAE type. Various variants in different genes including the factor XII *(F12),* the plasminogen (*PLG*), angiopoietin-1 (*ANGPT1*) and kininogen-1 (*KNG1*) genes were identified in patients of large families with HAEnCI across 3 or more generations and were assumed to be involved in the development of types of HAE [5–10] (Table 1).

**Table 1:** Types of hereditary angioedema with normal C1-INH

HAE type	Gene	Nucleotide change	AA change	Chromosome	Variant first described
HAE-FXII	*F12*	c.983C > A	p.T328K	5	Dewald and Bork 2006 [5]
HAE-FXII	*F12*	c.983C > G	p.T328R	5	Dewald and Bork 2006 [5]
HAE-FXII	*F12*	c.971_1018 + 24del72	Indel	5	Bork et al. 2011 [11]
HAE-FXII	*F12*	c.892_909dup	Duplication p.298_303	5	Kiss et al. 2013 [12]
**HAE-PLG**	***PLG***	**c.988A > G**	**p.K330E**	**6**	**Bork et al 2018 [8]**
HAE-ANGPT1	*ANGPT1*	c.807G > T	p.A119S	8	Bafunno et al. 2018 [7]
HAE-KNG1	*KNG1*	c.1136 T > A	p.M379K	3	Bork et al. 2019 [9]

*AA* amino acid, *ANGPT1* angiopoietin-1, *F12* coagulation factor XII gene, *FXII* coagulation factor XII protein, *HAE* hereditary angioedema, *HAE-PLG* HAE with the c.988A > G (p.K330E) variant in the plasminogen gene; *KNG1* kininogen1 gene

One of these types is “HAE with the c.988A>G (p.Lys330Glu; p.K330E) variant in the *PLG* gene” or HAE-PLG. It was described in 13 German families [8]. The basic genetic alteration is the missense variant c.988A > G leading to the amino acid exchange p.K330E (identical to position p.K311E if numbering excludes the signal peptide of 19 amino acids) in the kringle 3 domain in the plasminogen protein [8]. After first identification of the basic genetic alteration in the *PLG* gene by whole exome sequencing [8] it is now easily possible to determine the HAE-PLG variant using genetic standard methods (Sanger sequencing). Until now, additional patients with HAE-PLG were identified in Germany and various other European countries, in Japan, and in the US [13–16].

In the first report about HAE-PLG it was shown that tongue swelling is a common symptom [8]. We reported about 3.795 tongue swellings in 47 patients. Among these, 331 tongue swellings in 23 patients were associated with dyspnoea, voice changes and imminent asphyxiation. Two patients asphyxiated due to tongue swellings that lead to obstruction of the upper airways [8]. Another patient with HAE-PLG was reported to have died from asphyxiation [16]. Since swelling of the tongue is potentially life-threatening, it is important to identify effective treatments for patients with HAE-PLG. Therefore, the aim of this observational, retrospective study was to analyze our data on a high number of attacks treated with on-demand as well as with long-term prophylaxis and to compare different treatment options for this rare condition. These findings may help to elucidate further the pathways and “mediators” involved in the formation of attacks in patients suffering from HAE-PLG.

## Results

The total cohort consisted of 111 symptomatic individuals coming from 22 families with the *PLG* gene variant c.988A > G (p.K330E). All patients had a confirmed diagnosis of HAE-PLG according to the first description of a novel variant of the *PLG* gene in 2017 [8]. Before 2017, patients were classified as having HAEnCI and an unknown genetic background (HAE-unknown) or idiopathic angioedema; after 2017 they were re-diagnosed as HAE-PLG. Eight additional family members were symptom-free carriers of the *PLG* variant K330E, i.e. never had angioedema symptoms. A total of 59/111 patients had received treatments for acute attacks or for long-term prophylaxis (LTP) of HAE-PLG and 52/111 symptomatic patients had never received any treatment for HAE. Baseline characteristics and laboratory results of the 59 patients who had received any treatment for HAE-PLG, are listed in Table 2. In all patients, C1-INH activity, C1-INH protein, and C4 in plasma were normal. Plasminogen activity in plasma during the attack-free interval, obtained from 34 patients, was similar (91%; SD 17.4%) to that of a control group of 30 healthy individuals (93.1%; SD 14.2%, *p* < 0.60).

**Table 2:** Baseline characteristics

Characteristic	Treated patients with HAE-PLG *N* = 59*
Sex, n (%)	
Male	17 (28.8)
Female	42 (71.2)
Age, year	53.7 ± 17.1
Mean disease duration, year	21.4 ± 15.3
Laboratory parameters	
C1-INH activity (%) (RR 70–130)	99.4 ± 17.3
C1-INH protein (mg/dL) (RR 15.4–33.8)	24.8 ± 5.6
C4 (mg/dL) (RR 16.4–31.3)	24.0 ± 8.2
Plasminogen activity (%)	91 ± 17.4**

*C1-INH* C1 inhibitor, *HAE* hereditary angioedema, *HAE-PLG* HAE with the c.988A > G (p.K330E) variant in the *PLG* gene; *N* number of patients, *n* number of patients in the specified category, *RR* reference range, ± standard deviation

*Patients who had received treatment for acute attacks and/or long-term prophylaxis

**in 34 patients only; control group see text

### Treatment for acute attacks

#### Icatibant

Icatibant was used in 13 patients for 201 acute facial and abdominal attacks and tongue swellings. The mean duration of the treated attacks (mean 4.3 h; SD 2.6 h) was significantly shorter than that of the previous 149 untreated attacks (mean 44.7 h; SD 28.6 h, *p* < 0.0001). On average, administration of icatibant shortened the duration of swellings attacks by 88%. Icatibant was administered at home by 2 patients (for 129/133 attacks in 1 patient and 48/52 attacks in the other patient). Table 3 shows the mean duration of untreated and treated attacks per patients by attack location. Good response was seen in the vast majority of attacks (197/201). Low response was seen in 2 attacks in 1 patient and no response in 2 other patients with 1 attack each. On a per-patient basis, icatibant was effective in 11 and ineffective in 2 patients compared with the other 3 treatments pdC1-INH, corticosteroids alone and corticosteroids combined with antihistamines, as reported below (*p* < 0.01). On a per-attack basis, with icatibant treatment, there were significantly more attacks with good responses, and significantly fewer with low or no responses compared with the other treatments (*p* < 0.001). One patient had an initial improvement of an abdominal attack after treatment with icatibant and a recurrence of symptoms 9 h after the icatibant injection.

**Table 3:** Efficacy of icatibant in 201 swellings versus 149 untreated attacks in 13 patients with HAE-PLG

Patient number	No. of untreated attacks*	Mean duration of untreated attacks (hrs)	No. of treated attacks	Mean time between attack onset and injection of icatibant (hrs)	Mean time to first symptom relief (hrs)	Mean duration of treated attacks (hrs)	Mean shortening of attack duration attacks (%)	No. of attacks shortened by > 50%	No. of attacks shortened by 20–50%	No. of attacks shortened by < 20%
Facial attacks										
1	10	47.6	65	0.25	0.5	4.2	91.2	64	1	0
2	10	105.1	2	3.5	0.5	6.5	93.8	2	0	0
3	10	59.9	1	3	1	17	71.6	1	0	0
Tongue swellings										
1	10	36.4	41	0.25	0.5	4	89	40	1	0
4	10	17.3	1	1	0.5	6	65.3	1	0	0
5	10	35.1	52	1	0.25	4.3	87.7	52	0	0
6	10	13.3	1	3	7	13	2.3	0	0	1
7	10	30.2	2	1	0.5	3	90.1	2	0	0
8	6	70.2	2	2	0.3	2.5	96.4	2	0	0
9	3	37.7	1	0.5	0.5	3	92	1	0	0
10	10	3.1	1	0.3	0.3	3.1	1	0	0	1
11	10	31.1	1	0.5	0.2	2	93.6	1	0	0
12	10	55.9	1	1	0.5	4	92.8	1	0	0
13	10	72.5	1	0.5	0.5	1	98.6	1	0	0
Abdominal attacks										
1	10	27.7	27	0.2	0.5	4.4	84.1	27	0	0
3	10	77.9	2	1	0.5	1.5	98.1	2	0	0
Total No.	149	–	201	–	–	–	–	197	2	2

*C1-INH* C1 inhibitor, *HAE* hereditary angioedema, *HAE-PLG* HAE with normal C1-INH and the c.988A > G (p.K330E) variant in the plasminogen gene; No. = number

*Last 10 attacks before treatment or all if less than 10

Note: Out of the 201 swellings, 68 were facial swellings, 104 tongue swellings, and 29 abdominal attacks

#### Plasma-derived C1-INH

Plasma-derived (pdC1-INH) was used in 12 patients for 74 acute facial attacks, abdominal attacks or tongue swellings. The mean duration of the treated attacks (mean 31.5 h; SD 18.6 h) was significantly shorter than that of the previous 129 untreated attacks (mean 48.2 h; SD 32.5 h, *p* < 0.0001). On average, administration of pdC1-INH decreased attack duration by 44%. On a per-patient basis, pdC1-INH was effective in 7, and ineffective in 5 patients. On a per attack-basis, with pdC1-INH treatment, there were significantly more attacks with good responses than with low responses but also more attacks with no response compared with the other treatments (*p* < 0.05). Home treatment with pdC1-INH by a caregiver was used in 3 patients (for 17/32 attacks in 1 patient, 4/5 attacks in the second and 9/10 attacks in the third patient). Table 4 shows the mean duration of untreated and treated attacks per patients by attack location. Good response was seen in 29 attacks in 9/12 patients. A total of 12/29 attacks were treated with 500 IU, 16/29 with 1000 IU, and 1/29 attack with 1500 IU. PdC1-INH treatment resulted in low or no response in 45 attacks in 8 patients. A total of 9/45 attacks were treated with 500 IU, 34/45 with 1000 IU, 1/45 with 1500 IU and 1/45 with 3000 IU. In 2 patients, 3 tongue swellings progressed to a severe stage, despite treatment with pdC1-INH.

**Table 4:** Efficacy of pdC1-INH in 74 swellings versus 129 untreated attacks in 12 patients with HAE-PLG

Patient number	No. of untreated attacks*	Mean duration of untreated attacks (hrs)	No. of treated attacks	No. of attacks treated with 500 IU/1000 IU/1500 IU/3000 IU pdC1-INH	Mean time between attack onset and injection of icatibant (hrs)	Mean time to first symptom relief (hrs)	Mean duration of treated attacks (hrs)	Mean shortening of attack duration attacks (%)	No. of attacks shortened by > 50%	No. of attacks shortened by 20–50%	No. of attacks shortened by < 20%
Facial attacks											
1	10	47.6	20	4/16/0/0	1.7	2.6	45.3	4.8	0	0	20
2	10	105.1	6	6/0/0/0	3.3	1	18	82.9	6	0	0
14	10	67.1	1	0/1/0/0	2.5	1	24	64.2	1	0	0
Tongue swellings											
1	10	36.4	3	0/3/0/0	4	5	30	17.6	0	0	3
4	10	17.3	4	2/0/1/1	1	0.3	54.5**	0	0	0	4
15	10	2.7	1	1/0/0/0	2	0.5	1	62.7	1	0	0
16	10	34.2	6	6/0/0/0	2	1	12	64.5	5	1	0
17	3	48	2	0/1/1/0	6	1	21	56.3	2	0	0
18	6	96	2	0/2/0/0	0.5	0.2	12	87.5	2	0	0
19	10	24.4	1	0/1/0/0	2	1	6	75.4	1	0	0
20	10	20.8	1	0/1/0/0	2	24	48**	0	0	0	1
Abdominal attacks											
1	10	27.7	12	2/10/0/0	0.2	3.6	26.3	5.1	0	2	10
21	10	84.3	5	0/5/0/0	24	0.4	60.2	28.6	3	0	2
22	10	81.9	10	0/10/0/0	2.5	1	22.2	72.9	8	2	0
Total No.	129	–	74	21/50/2/1	–	–	–	–	29	5	40

*HAE* hereditary angioedema, *HAE-PLG* HAE with normal C1-INH and the c.988A > G (p.K330E) variant in the plasminogen gene; *pdC1-INH* plasma-derived C1-INH, *No.* number

*Last 10 attacks before treatment or all if less than 10

**Severe and rapidly developing attacks

Note: Out of the 74 swellings, 27 were facial swellings, 20 tongue swellings, and 27 abdominal attacks

#### Corticosteroids and antihistamines

**Fig. 1:**
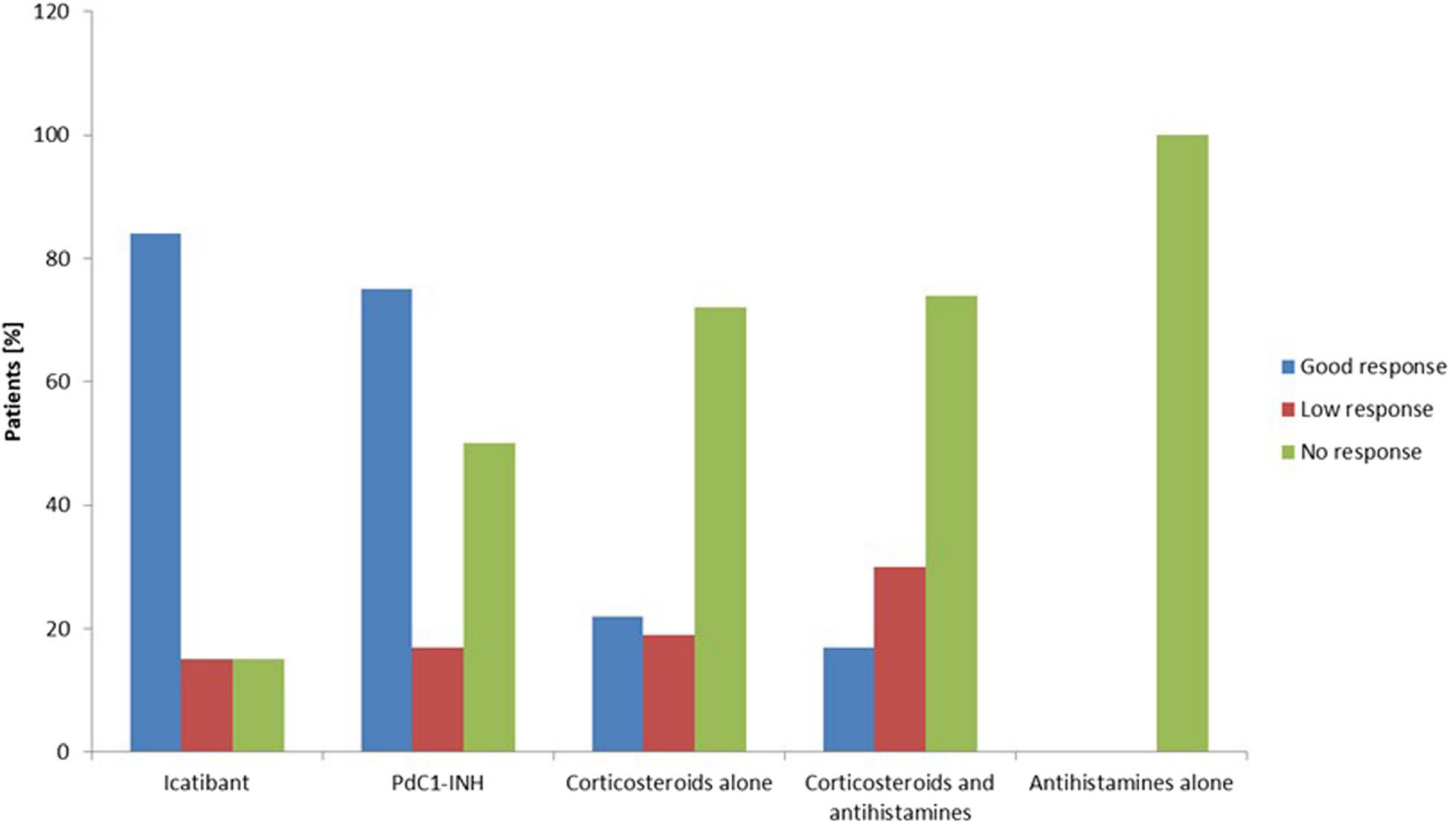
Treatment response of various treatments (per-patient analysis) in patients with HAE-PLG C1-INH=C1 inhibitor; HAE = hereditary angioedema; HAE-PLG = HAE with normal C1-INH and the c.988A > G (p.K330E) variant in the *PLG* gene; pdC1-INH = plasma-derived C1 inhibitor. Note: 13 patients were treated with icatibant, 12 patients with pdC1-INH, 36 patients with corticosteroids alone, 23 patients with corticosteroids and antihistamines in combination, and 5 patients with antihistamines alone. Percentages of patients do not sum up to 100% because patients could be classified to more than one response category

**Fig. 2:**
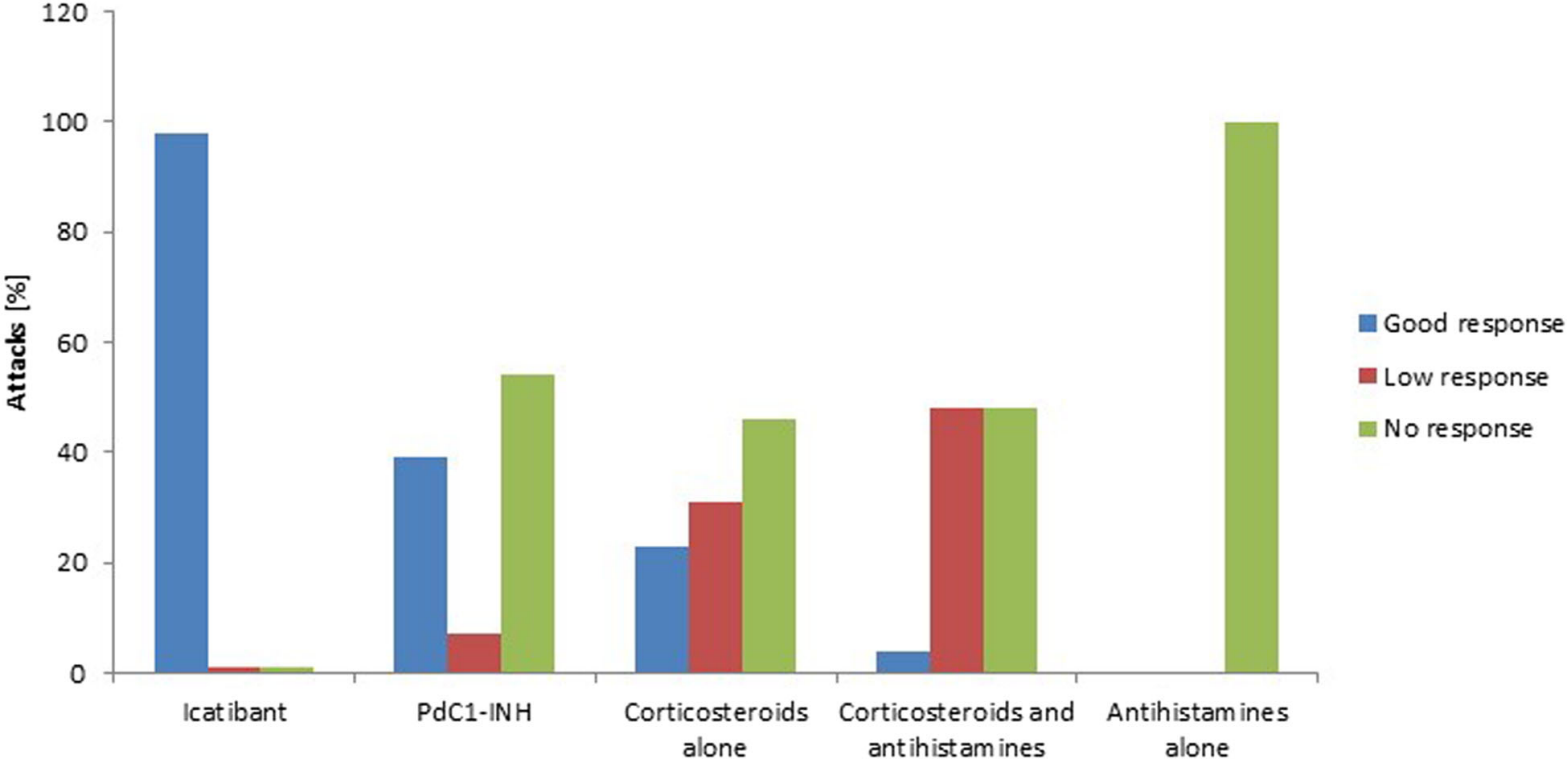
Treatment response of various treatments (per attack analysis) in % of acute attacks in HAE-PLG patients C1-INH=C1 inhibitor; HAE = hereditary angioedema; HAE-PLG = HAE with normal C1-INH and the c.988A > G (p.K330E) variant in the *PLG* gene; pdC1-INH = plasma-derived C1 inhibitor. Note: 201 attacks were treated with icatibant, 74 attacks with pdC1-INH, 268 attacks with corticosteroids alone, 309 attacks with corticosteroids plus antihistamines, and 37 attacks with antihistamines alone

Despite the fact that corticosteroids and antihistamines have limited or no value in bradykinin-mediated angioedema, they are sometimes used by physicians as a probatory treatment. A total of 53 patients received corticosteroids for 577 acute HAE-PLG attacks. 36 patients received corticosteroids alone for a total of 268 attacks, 23 patients received a combination of corticosteroids and antihistamines for 309 attacks, and 5 patients received antihistamines alone for 37 attacks. Corticosteroids alone showed high efficacy in 61/268 attacks in 8 patients, low efficacy in 82/268 attacks in 7 patients, and no efficacy in 125/268 attacks in 26 patients (Figs. 1 and 2). On a per-patient basis, corticosteroids alone were effective in 9 patients and ineffective in 27 patients. On a per-attack basis, with corticosteroids alone, there were significantly fewer attacks with good responses (*p* < 0.001) and significantly more attacks with poor or no responses (*p* < 0.01) than with the other treatments. Five of the 8 patients with high efficacy reported that treated swelling attacks developed more slowly and were clearly shorter than untreated attacks. One patient reported that oral corticosteroids given early in the attack were clearly effective in 5 lip swellings. Corticosteroids combined with antihistamines showed a high efficacy in 13/309 attacks in 4 patients, low efficacy in 150/309 attacks in 7 patients, and no efficacy in 146/309 attacks in 17 patients. On a per-patient basis, corticosteroids combined with antihistamines were effective in 5 patients and ineffective in 18 patients. On a per-attack basis, treatment with corticosteroids combined with antihistamines resulted in significantly fewer attacks with good responses (*p* < 0.001), and significantly more with low or no responses (*p* < 0.01) compared with the other treatments. Antihistamines alone were ineffective in all 37 attacks of 5 patients.

#### Epinephrine in combination with corticosteroids and antihistamines

A total of 5 patients received epinephrine aerosol for 11 severe tongue swellings. In these patients epinephrine was not given as a single drug but together with corticosteroids alone (9 tongue swellings) or combined with corticosteroids and antihistamines. Physicians and patients reported about slow or rapid improvement.

#### Fresh frozen plasma

In 2 patients with imminent asphyxiation due to tongue swellings and partial obstruction of the upper airways, fresh frozen plasma (FFP) was used. One patient received 1000 mL and the other 800 mL. In both patients FFP could not halt the progression of the attacks.

### Long-term prophylaxis

A total of 14 patients received LTP with either progestins, tranexamic acid (TXA), danazol, corticosteroids or antihistamines. Table 5 shows that LTP with desogestrel was very effective in 2/6 women who previously discontinued oral contraceptives. It was partially effective in 1/6 women, and ineffective in 3/6 women. The attack frequency was reduced by 46%. Three patients were treated with TXA for a total of 29 patient years; annual attack rate was reduced by 80 to 100% (mean attack reduction of 94%). Three patients received danazol for periods ranging from 4 months to 8 years. Two patients had no attacks and 1 patient had 2 attacks under danazol. The attack rate was reduced by a mean of 83%. Two patients received a LTP with corticosteroids for 5 years and 4 weeks, respectively, and 2 further patients received antihistamines for 5 months (fexofenadine) and 3 months (loratadine), respectively. These treatments were ineffective in prevention or reduction of attack rate.

**Table 5:** Attack frequency in patients with HAE-PLG before and during long-term prophylaxis

Patient No.	Medication	Symptomatic years before LTP	Attacks during symptomatic years before LTP	Years with LTP	Attacks during LTP	Reduction in attack frequency (%)
Progestins*						
1	desogestrel	4	75	4	82	0
5	desogestrel	7	432	2	75	0
15	desogestrel	12	135	2	26	0
23	desogestrel	7	83	1	0	100
24	desogestrel	10	210	3	14	77.6
25	desogestrel	6	10	2	0	100
Antifibrinolytics						
20	TXA	69	829	4	0	100
26	TXA	7	13	14	0	100
27	TXA	15	17	11	2	81.8
Attenuated androgens**						
13	danazol	13	461	8	0	100
20	danazol	58	698	0.3	2	50
27	danazol	15	17	0.3	0	100

*C1-INH* C1 inhibitor, *HAE* hereditary angioedema, *HAE-PLG* HAE with normal C1-INH and the c.988A>G (p.K330E) variant in the *PLG* gene, *LTP* long-term prophylaxis, *TXA* tranexamic acid

*after discontinuing estrogen-containing oral contraceptives

**dose range 100 mg to 200 mg danazol daily

## Discussion

Recurrent angioedema without urticaria is a symptom of several disease entities. If recurrent angioedema occurs in 2 or more family members with normal C1-INH a diagnosis of HAEnCI can be suspected. Since no confirmatory diagnostic plasma tests exist for the various types of HAEnCI, genetic testing will eventually lead to the diagnosis of HAE-PLG, which is a potentially life-threatening condition because asphyxiation due to an acute obstruction of the upper airways is not uncommon. Due to this risk and overall burden of disease, comprehensive care of patients with HAE-PLG is necessary [17], as with all other types of HAE. So far, there are only a few case series of affected patients with HAE-PLG that have been reported. The prevalence of HAE-PLG is not known at present but seems to be much lower than of HAE-C1-INH. There is also only very limited information about the different treatments used in HAE-PLG patients [8, 13, 14]. Randomized controlled double-blind studies are not available at present and are unlikely to be conducted since this is an ultra-rare condition. Also, treatment with placebo cannot be justified, as any attack in these patients may be fatal and therefore all need to be effectively treated.

In the present study, we could demonstrate that various treatments currently used to treat acute swelling attacks and those used as prophylactic agents were generally effective and prevented death from asphyxiation in all patients with HAE-PLG. However, it appears that some treatments were more effective than others. Treatment with icatibant for acute attacks turned out to be effective in nearly all patients with HAE-PLG and in over 90% of acute attacks. Icatibant is a short-lived bradykinin B2 receptor antagonist blocking the effects of bradykinin at the receptor level [18]. Icatibant has been shown to be highly effective in the treatment of angioedema attacks of HAE-C1-INH, with a high response rate of attacks and a rapid response [19]. Swelling attacks in HAE-C1-INH are due to an uncontrolled activation of the contact system/kallikrein-kinin system (KKS), with an overproduction of the vasoactive bradykinin [20]. Response rates to icatibant are similarly high in both HAE-C1-INH and HAE-PLG. As icatibant is a B2 receptor antagonist and Lys-bradykinin is an important ligand for B2 receptor this suggests that bradykinin is the major mediator in both conditions. An overproduction of bradykinin has never been directly shown in HAE-PLG patient samples, also not by evaluation of high molecular weight kininogen cleavage products during attacks. However, our observation of good treatment response to icatibant serves as an indirect demonstration for bradykinin accumulation as the main pathophysiological cause for angioedema symptoms in HAE-PLG. In HAE-PLG, it is known that the variant in the *PLG* gene leads to an amino acid exchange in the kringle 3 domain of plasminogen. The kringle 3 domain serves for attachment of plasminogen on the cell surface [21, 22]. The consequence might be an increased activation of the fibrinolytic system with subsequent formation of plasmin, activation of KKS, and increased production of bradykinin [8].

The present study showed normal values for plasminogen in plasma during the attack-free interval. This seems to indicate that the *PLG* gene variant of HAE-PLG has no influence on plasminogen activity in blood plasma. Plasminogen is a zymogen that cannot support any biological function unless it is converted into plasmin by the 2 main plasminogen activators urokinase and tissue plasminogen activator. Other phenotypes linked to other variants in the *PLG* gene are hypoplasminogenemia and dysplasminogenemia [23, 24]. The described patients had low plasminogen activity in plasma but angioedema was not reported.

According to our observations, pdC1-INH was very effective for the treatment of a high number of acute attacks in the majority of patients with HAE-PLG. However, in some patients and a number of attacks, pdC1-INH was less effective or even ineffective. This is in contrast to HAE-C1-INH, where treatment with pdC1-INH is usually promptly and consistently effective in almost all patients [25]. This is an interesting observation, since patients with HAE-PLG do not show C1-INH deficiency between attacks. C1-INH is a strong inhibitor of kallikrein and controls the KKS activation. It is suggested that C1-INH is consumed at the start of an acute attack which is then causing an uncontrolled activation of KKS. This relative overconsumption could be at least partially compensated by treatment with pdC1-INH.

In HAE-C1-INH and acquired angioedema due to C1-INH deficiency, which are both mediated by bradykinin, treatment with corticosteroids and antihistamines is not expected to be effective. The results of the present study show that acute attack treatment with antihistamines alone is indeed ineffective in HAE-PLG patients, as analyzed on a per-patient and per-attack basis. Treatment for acute attacks of HAE-PLG with corticosteroids, however, may have a certain benefit, at least in some patients and some attacks, while the majority of patients does not respond at all. From a pathophysiologic point of view there is currently no understanding of why corticosteroids might be effective in some HAE-PLG patients and for some attacks. Nevertheless, this medication is sometimes used by physicians as a probatory treatment. We cannot recommend corticosteroids as a primary treatment option for HAE-PLG attacks.

The effectiveness of long-term treatment with progestins after discontinuing oral contraceptives was found to be in a range of no to complete prevention of further attacks. The effectiveness of progestins appears to be not specific to HAE-PLG but has also been observed in patients with HAE-C1-INH, HAE linked with a variant in the *F12* gene (HAE-FXII) and idiopathic angioedema [26, 27]. The exact mode of action of progestins in those types of angioedema is unknown.

We treated 3 patients with HAE-PLG with TXA for a total of 29 patient years and observed good to excellent efficacy. This supports the assumption that fibrinolysis is involved in the pathogenesis of HAE-PLG. Plasmin can activate the KKS and may thus lead to bradykinin formation [28]. The activation can be partially or completely blocked by TXA, which could explain the clinical efficacy of TXA in HAE-PLG.

Danazol belongs to the 17-alpha alkylated androgens that have been effective in HAE-C1-INH and also HAE-FXII. Our results of danazol demonstrate a high effectiveness in 3 patients with HAE-PLG treated for a total of 8.6 years.

The main limitation of our study is that it is a retrospective observational study and patients have been assigned to their respective treatment based on discretion by the treating physician. No prospective, randomized placebo-controlled double-blind study has been performed so far in patients having this ultra-rare condition. But information about treatment experience is warranted and important to be communicated, as asphyxiation is not uncommon in HAE-PLG.

## Conclusions

Given the limitations mentioned above, the results of this relatively large patient cohort show that there are various treatment options available that are able to reduce symptoms in patients with HAE-PLG either completely or at least partially. Considering the limited number of treated patients and attacks of HAE-PLG, our results support the use if icatibant as first line treatment for acute attacks, followed by pdC1-INH concentrate. Corticosteroids and antihistamines cannot be recommended due to the high number of non-responders. For LTP, TXA can be recommended as first line treatment. The use of attenuated androgens is limited for LTP due to the well-known risk of side effects.

## Methods

### Patients

Patients for this retrospective, observational study were followed up at the Angioedema Outpatient Service, Department of Dermatology, University Medical Center Mainz, Germany from January 1999 to July 2019. All patients had a confirmed diagnosis of HAE-PLG according to the first description of a novel variant of the *PLG* gene in 2017 [8]. Before, these patients had been classified as having HAEnCI and an unknown genetic background (HAE-unknown) or idiopathic angioedema. Diagnosis of HAE-PLG was based on personal history of recurrent angioedema, positive family history of angioedema, plasma examination of C1-INH, C4, and C1q, and genetic testing. All plasma samples from patients with HAE-PLG were drawn during the symptom-free interval between attacks. The study was approved by the local ethics committee (Ethics Committee of the Landesärztekammer Rheinland-Pfalz, 837.413.13 [9098-F]) and all patients gave their informed consent to participate in the study.

### Study design

The present study is a retrospective, observational study. The patient cohort consisted of a total of 111 patients who presented to the outpatient clinic with angioedema symptoms. A total of 59/111 patients reported about the efficacy of various treatments for HAE-PLG. For acute attacks 58 patients had received HAE-specific medication (icatibant and/or pdC1-INH) or non-HAE-specific medication (corticosteroids, antihistamines, epinephrine) or FFP. For LTP, patients had received desogestrel, TXA, danazol, corticosteroids or antihistamines. These medications were generally administered to treat different types of angioedema and had been used in the patients in this study due to a suspected bradykinin- or histamine-mediated cause of their symptoms. The choice of treatment had been based solely at the physician’s discretion and no specific treatment algorithm was applied.

Patients on icatibant and/or pdC1-INH had recorded their attack symptoms (location, attack duration, severity and treatment) in a patient diary. The treatment effect was assessed by an intra-individual comparison of the attack duration of treated versus untreated attacks. Patients who had received non-HAE-specific medication for acute attacks were evaluated by their referring physicians’ and main treatment outcomes (data about hospital stays for HAE attacks and attack characteristics [response to treatment and attack frequency] were collected retrospectively by patient questionnaires).

Efficacy outcomes were assessed qualitatively as good response (over 50% reduction of attack duration), low response (20–50% reduction of attack duration), and no response (< 20% reduction of attack duration).

Data from 14 patients who had received LTP were obtained in a similar way. The efficacy of LTP was assessed by an intra-individual comparison of the number of attacks before and during LTP normalized by duration of observation period and calculated as a mean % attack reduction.

### Laboratory and statistical methods

C1-INH function was determined using the chromogenic substrate C_2_H_5_CO-Lys(ε-Cbo)-Gly-Arg-pNA (Immunochrom C1-INH, Technoclone, Vienna, Austria). Antigenic levels of C1-INH, and C4 were quantified by radial immunodiffusion. Plasminogen activity was determined using a chromogenic assay: Plasminogen is activated through reaction with an excess of Streptokinase in the presence of fibrinogen. The plasminogen-Streptokinase complex is determined by the rate of hydrolysis of the chromogenic substrate pyroGlu-Phe-Lys-pNA (HemosIL Plasminogen, Instrumentation Laboratory, Bedford, Mass., USA) [29]. The presence of the c.988A > G (p.K330E) *PLG* gene variant was tested as described elsewhere [8]. Assessing significant differences, t-tests and chi-square tests at α = 0.05 (2-sided) were applied using STATA (version 12, StataCorp, College Station, Texas, USA).

## Data Availability

The datasets used and/or analyzed during the current study are available from the corresponding author on reasonable request.

## Abbreviations

C1-INH: C1 inhibitor
FFP: Fresh frozen plasma
HAE: Hereditary angioedema
HAE-C1-INH: HAE due to C1-INH deficiency
HAE-FXII: HAE linked with variants in the *F12* gene
HAEnCI: HAE with normal C1-INH
HAE-PLG: HAE with the c.988A > G (p.K330E) variant in the plasminogen gene
KKS: Kallikrein-kinin system
LTP: Long-term prophylaxis
pdC1-INH: Plasma-derived C1-INH
PLG: Plasminogen
SD: Standard deviation
TXA: Tranexamic acid
